# [(1,2,5,6-η)-Cyclo­octa-1,5-diene](1-ethyl-4-isopropyl-1,2,4-triazol-5-yl­idene)(tri­phenylphos­phane)iridium(I) tetra­fluorido­borate di­chloro­methane sesquisolvate

**DOI:** 10.1107/S2414314623009033

**Published:** 2023-10-19

**Authors:** Aaron Maynard, Michael Gau, Daniel R. Albert, Edward Rajaseelan

**Affiliations:** a Department of Chemistry, Millersville University, Millersville, PA 17551, USA; b Department of Chemistry, University of Pennsylvania, Philadelphia, PA 19104, USA; University of Aberdeen, United Kingdom

**Keywords:** crystal structure, iridium, N-heterocyclic carbenes

## Abstract

The synthesis and crystal structure of a new triazole-based N-heterocyclic carbene iridium(I) cationic complex with a tetra­fluorido­borate counter-anion and solvating di­chloro­methane, [Ir(C_8_H_12_)(C_7_H_13_N_3_)(C_18_H_15_P)]BF_4_·1.5CH_2_Cl_2_, is reported. The Ir^I^ center of the cationic complex has a distorted square-planar conformation, formed by a bidentate cyclo­octa-1,5-diene (COD) ligand, an N-heterocyclic carbene, and a triphenylphosphane ligand. There are weak hydrogen-bonding inter­actions between C—H groupings of the iridium complex and F atoms of the [BF_4_]^−^ counter-ions. The atoms of the COD ligand are disordered over two sets of sites in a 0.65:0.35 ratio and two of the F atoms of the anion are disordered over adjacent sites in a 0.6:0.4 ratio. One of the di­chloro­methane solvent mol­ecules is disordered about an inversion center with 0.5 occupancy.

## Abstract 2

The synthesis and crystal structure of a new triazole-based N-heterocyclic carbene iridium(I) cationic complex with a tetra­fluorido­borate counter-anion and solvating di­chloro­methane is reported. The IrI center of the cationic complex has a distorted square-planar conformation, formed by a bidentate cyclo­octa-1,5-diene (COD) ligand, an N-heterocyclic carbene, and a tripheylphosphane ligand. There are weak hydrogen-bonding inter­actions between C—H groupings of the iridium complex and F atoms of the [BF_4_]^−^ counter-ions.

## Graphical abstract

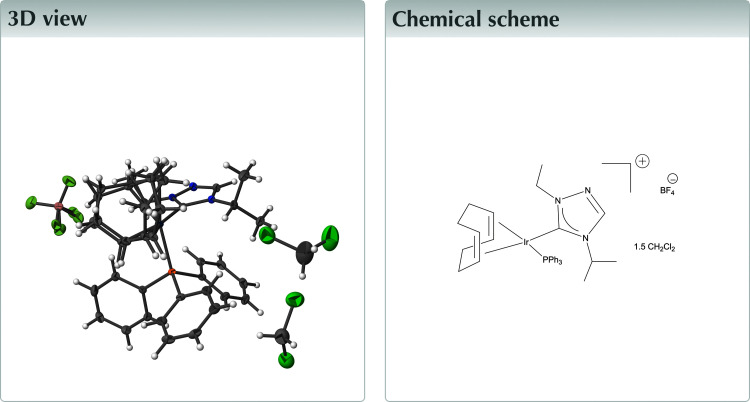

## Structure description

N-heterocyclic carbenes (NHCs) have emerged as excellent supporting ligands in late transition-metal catalysis (Cazin, 2013 ▸; de Frémont *et al.*, 2009 ▸; Díez-Gonzáles *et al.*, 2009 ▸; Rovis & Nolan, 2013 ▸; Ruff *et al.*, 2016 ▸; Zuo *et al.*, 2014 ▸). They have shown catalytic activity in the transfer hydrogenation of ketones and imines (Albrecht *et al.*, 2002 ▸; Gnanamgari *et al.*, 2007 ▸). The NHC ligands can be tuned sterically and electronically by having different substituents on the nitro­gen atoms (Gusev, 2009 ▸). Many imidazole- and triazole-based NHC rhodium and iridium complexes have been synthesized and structurally characterized (Herrmann *et al.*, 2006 ▸; Wang & Lin, 1998 ▸; Chianese *et al.*, 2004 ▸). We continue to synthesize new imidazole- and triazole-based NHC complexes, to study the effect of metals, different substituents on the NHCs, and the ancillary ligands coordinating to the metal in transfer-hydrogenation reactions (Maynard *et al.*, 2023 ▸; Nichol *et al.*, 2009 ▸, 2010 ▸, 2011 ▸, 2012 ▸; Idrees *et al.*, 2017*a* ▸,*b* ▸; Rood *et al.*, 2021 ▸; Rushlow *et al.*, 2021 ▸, 2022 ▸; Newman *et al.*, 2021 ▸; Castaldi *et al.*, 2021 ▸).

**Figure 1:**
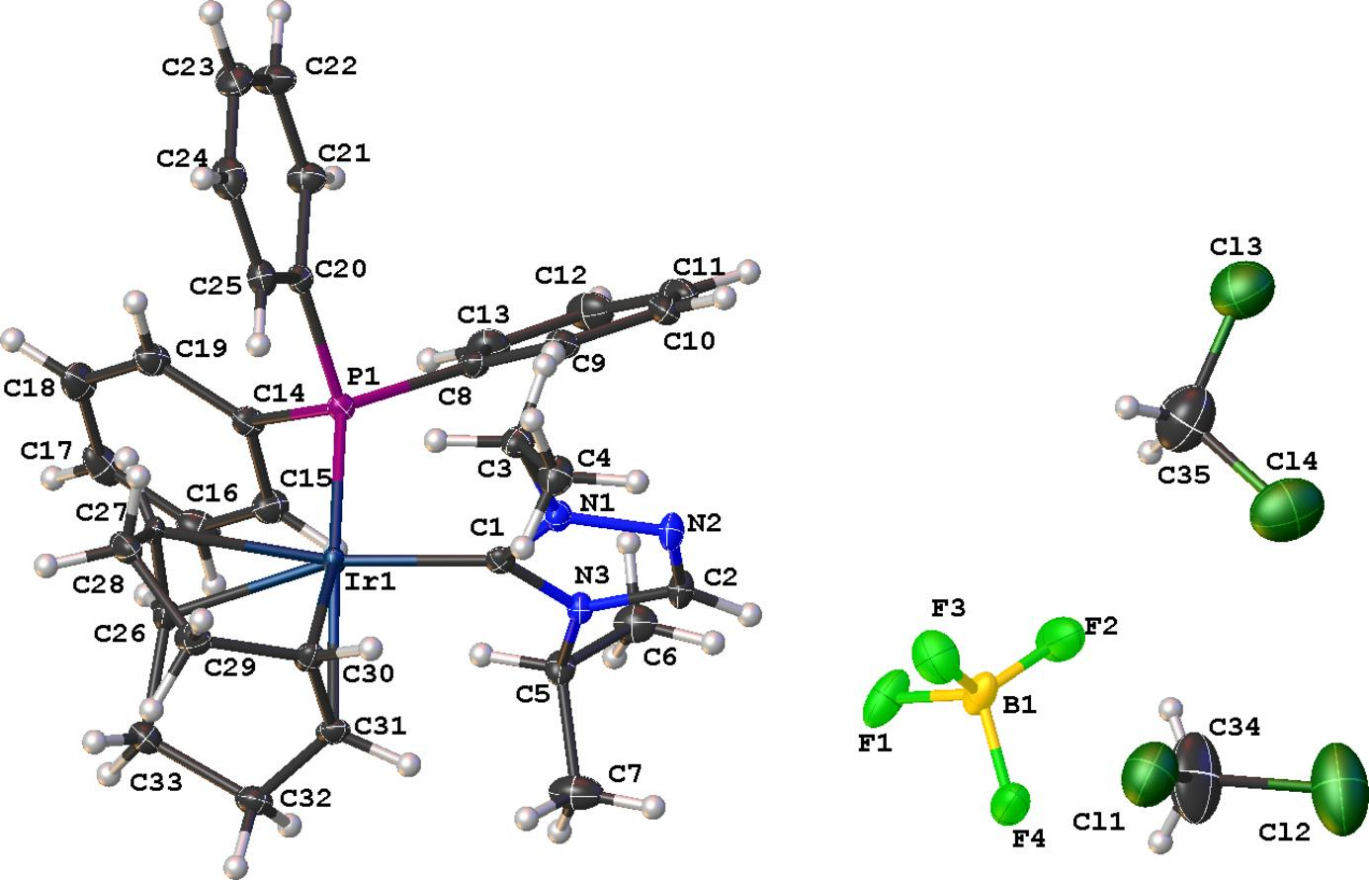
The mol­ecular entities in the crystal structure of the title compound **3**. Displacement ellipsoids are drawn at the 50% probability level.

The mol­ecular structure of the title complex (**3**), shown in Fig. 1 ▸, is characterized as an Ir^I^ cationic complex with a tetra­fluorido­borate counter-ion and incorporates 1.5 di­chloro­methane solvent mol­ecules. The distorted square-planar geometry of the coordination sphere around the Ir^I^ atom is formed by a bidentate cyclo­octa-1,5-diene (COD) ligand, the carbene C atom of the triazole NHC ligand, and the P atom of the tri­phenyl­phosphane ligand. The distorted square-planar geometry exhibits a P1—Ir1—C1 bond angle of 92.69 (7)°. The carbene C atom bonded to the central Ir^I^ atom exhibits a bond angle that significantly differs from the expected *sp* ^2^ hybrid­ization with an N1—C1—N3 bond angle of 103.5 (2)°, as observed in similar structures. An intra­molecular C—H⋯π(ring) inter­action is observed between a hydrogen atom on the isopropyl wingtip of the NHC (H6*A*) ligand and a phenyl phosphane ring (C8–C13) with an H⋯centroid distance of 2.67 Å and a C—H⋯centroid angle of 168°.

**Figure 2:**
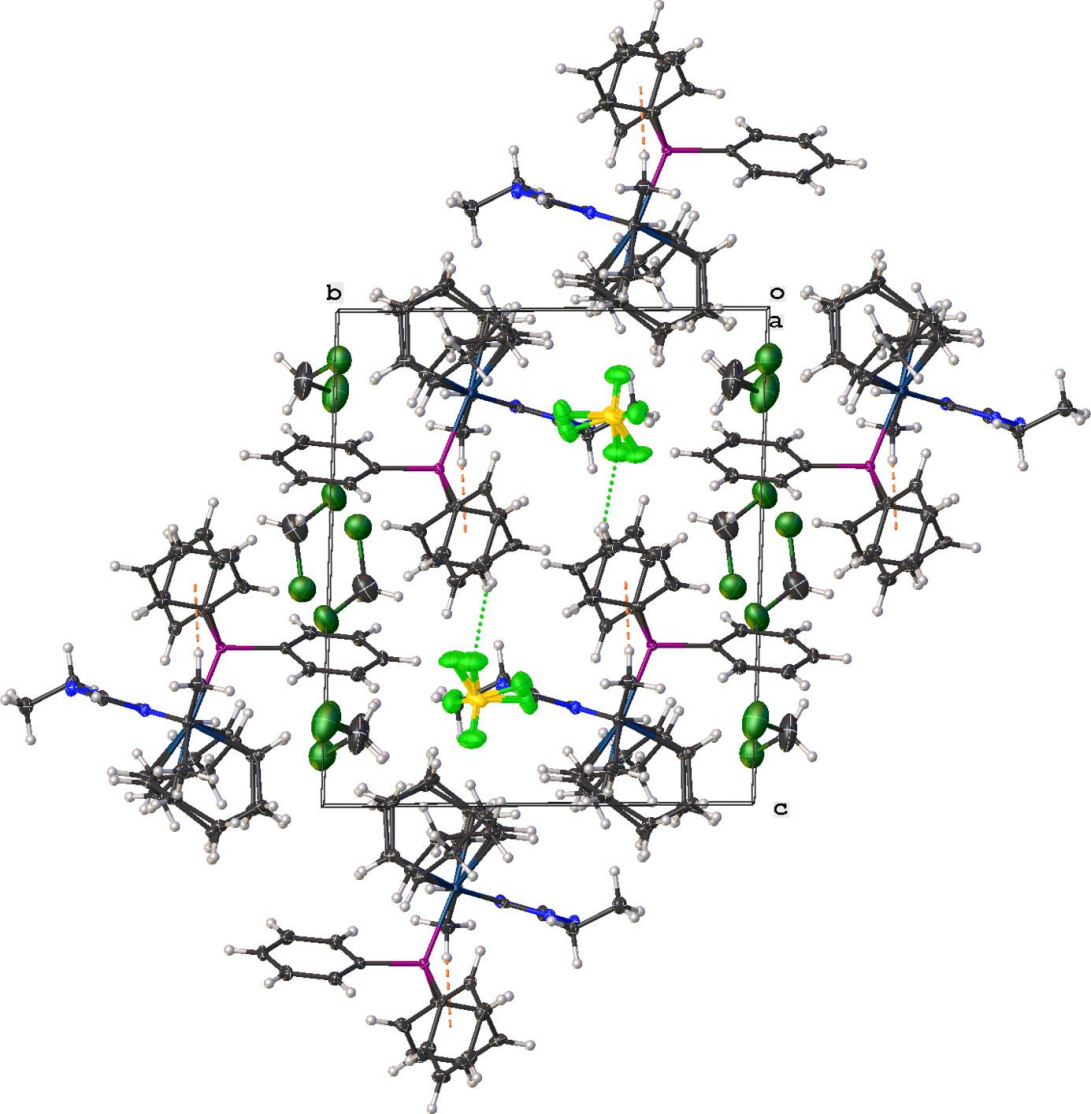
Crystal packing of **3** shown along the a axis direction. Non-classical hydrogen bonding inter­actions are shown as dotted green lines. C—H⋯π(ring) inter­actions are shown as dashed orange lines between hydrogen atoms and phenyl ring centroids.

In the extended structure, weak hydrogen-bonding inter­actions between a C—H grouping of the N-heterocyclic carbene and F atoms of the [BF_4_]^−^ counter-ion are observed along with inter­actions between the triphenyl phosphane ligand and the [BF_4_]^−^ counter-ion (Table 1 ▸). Fig. 2 ▸ shows the packing diagram of the title complex.

The crystal structure of the triazolium salt that was used in the synthesis of the title compound was previously determined (Maynard *et al.*, 2023 ▸). Comparison of triazolium salt bond angles and lengths with the bond angles and lengths of the NHC in the title complex are summarized in Tables 2 ▸ and 3 ▸, respectively. The most significant changes occur for the carbon atom coordinating to the metal center: the N1—C1—N3 bond angle goes from 107° in the triazolium salt to 103.5 (2)° when coordinating to the iridium atom as an NHC and the C—N bond lengths (C1—N1 and C1—N3) elongate by about 0.03 Å when the NHC coordinates to the metal.

## Synthesis and crystallization

**Figure 3:**
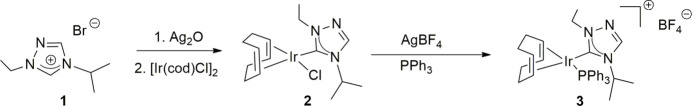
Reaction scheme for the synthesis of **3**.

**1-Ethyl-4-isopropyl-1,2,4-triazolium bromide (1)** was synthesized by a previously published procedure (Maynard *et al.*, 2023 ▸). All other compounds used in the syntheses as shown in Fig. 3 ▸ were obtained from Sigma–Aldrich and Strem and used as received. All subsequent synthesis procedures were performed under an N_2_ atmosphere using reagent grade solvents, which were used as received without further purification. NMR spectra were recorded at room temperature in CDCl_3_ on a 400 MHz (operating at 162 MHz for ^31^P) Varian spectrometer and referenced to the residual solvent peak (δ in ppm). The title compound (**3**) was crystallized by slow diffusion of pentane into a CH_2_Cl_2_ solution.

**[(1,2,5,6-η)-Cyclo­octa-1,5-diene](1-ethyl-4-isopropyl-1,2,4-triazol-5-yl­idene) chlorido­iridium (2).** 1-Ethyl-4-isopropyl-1,2,4 triazolium bromide (**1**) (0.065 g, 0.300 mmol), Ag_2_O (0.035 g, 0.149 mmol), and 10 ml of CH_2_Cl_2_ were added to an oven-dried flask and stirred under an N_2_ atmosphere in the dark for 90 min. The mixture was filtered through Celite into [Ir(cod)Cl]_2_ (0.100 g, 0.149 mmol) and stirred under dark for 90 min. The resulting mixture was filtered through Celite and the solvent was removed under reduced pressure. The orange solid product was washed with pentane and allowed to dry overnight under vacuum. Yield: 0.121 g (85.2%). ^1^H NMR: CDCl_3_, δ (ppm) 7.86 (*s*, 1 H, N—C3H—N), 4.69 [*m*, 1 H, CH(CH_3_)_2_], 4.33, 4.29 (*m*, 4 H, CH of COD) 4.12 (*q*, 2 H, CH_2_—N), 2.98, 2.21, 2.17, 1.79 (*m*, 8 H, CH_2_ of COD),1.02 [*m*, 6 H, CH(CH_3_)_2_], 0.874 (*t*, 3 H, CH_2_CH_3_). ^13^C NMR: δ 182.94 (Ir—C), 141.60 (N—CH—N), 86.40, 86.32 (CH of COD), 51.70 [CH(CH_3_)_2_], 39.60 (N—CH_3_), 33.42, 33.36, 29.62, 29.32 (CH_2_ of COD), 24.17, 23.31 [CH(CH_3_)_2_], 14.04 (CH_2_CH_3_).

**[(1,2,5,6-η)-Cyclo­octa-1,5-diene] (1-ethyl-4-isopropyl-1,2,4-triazol-5-yl­idene)(tri­phenyl­phosphane)iridium(I) tetra­fluor­ido­borate (3).** Tri­phenyl­phosphane (0.052 g, 0.198 mmol) and AgBF_4_ (0.038 g, 0.198 mmol) were added to an oven-dried flask containing complex **2** (0.094 g, 0.198 mmol) in 10 ml of CH_2_Cl_2_, and stirred under an N_2_ atmosphere in the dark for 90 min. The mixture was filtered through Celite, and the solvent was removed under reduced pressure. The bright orange–red solid was washed with pentane and dried under vacuum. Yield: 0.135 g (86.5%). ^1^H NMR: CDCl_3_, δ (ppm) 8.19 (*s*, 1 H, N—C3*H*—N), 7.47–7.24 (*m*, 15H, H_arom_), 5.30 [*m*, 1 H, CH(CH_3_)_2_], 4.45, 3.80 (*m*, 4 H, CH of COD), 4.35 (*m*, 2 H, N—CH_2_CH_3_), 2.61–1.61 (m,(CH_2_ of COD), 1.24 [*d*, 6 H, CH(CH_3_)_2_], 0.79 (*t*, 3H, N—CH_2_CH_3_). ^13^C NMR: δ 176.77 (Ir—C), 141.19 (N—CH—N), 133.74–128.42 (C_arom_), 87.18, 87.06, 85.47, 85.37 (CH of COD), 53.43 [CH(CH_3_)_2_], 47.71 (N—CH_2_), 32.29, 31.44, 29.89, 29.05 (CH_2_ of COD), 24.50, 22.09 [CH(CH_3_)_2_], 13.84 (N—CH_2_CH_3_). ^31^P: **δ** 17.78.

## Refinement

Crystal data, data collection, and structure refinement details are summarized in Table 4 ▸.

## Supplementary Material

Crystal structure: contains datablock(s) I. DOI: 10.1107/S2414314623009033/hb4453sup1.cif

Structure factors: contains datablock(s) I. DOI: 10.1107/S2414314623009033/hb4453Isup2.hkl

CCDC reference: 2301311

Additional supporting information:  crystallographic information; 3D view; checkCIF report

## Figures and Tables

**Table 1:** Hydrogen-bond geometry (Å, °)

*D*—H⋯*A*	*D*—H	H⋯*A*	*D*⋯*A*	*D*—H⋯*A*
C2—H2⋯F1^i^	0.95	2.21	3.140 (16)	168
C11—H11⋯F2^ii^	0.95	2.37	3.192 (13)	145

Symmetry codes: (i) 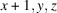 ; (ii) 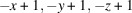 .

**Table 2:** Comparison of bond angles (°) for NHC and triazolium salt NHC data from this work. Triazolium salt data from Maynard *et al.* (2023 ▸).

Angle in NHC	NHC	Triazolium salt	Δ (salt to NHC)
N1—C1—N3	103.5 (2)	107.1	–3.6
N3—C2—N2	111.4 (2)	111.3	+0.1
C1—N3—C2	108.2 (2)	106.3	+1.9
C2—N2—N1	103.5 (2)	103.8	–0.3
N2—N1—C1	113.4 (2)	111.4	+2.0

**Table 3:** Comparison of bond lengths (Å) for NHC and triazolium salt NHC data from this work. Triazolium salt data from Maynard *et al.* (2023 ▸).

Bond in NHC	NHC	Triazolium salt	Δ (salt to NHC)
C1—N1	1.340 (2)	1.307	–0.033
C1—N3	1.368 (3)	1.336	–0.032
C2—N3	1.369 (3)	1.361	+0.008
C2—N2	1.304 (4)	1.306	–0.002
N1—N2	1.382 (3)	1.365	+0.017

**Table 4:** Experimental details

Crystal data	
Chemical formula	[Ir(C_8_H_12_)(C_7_H_13_N_3_)(C_18_H_15_P)]BF_4_·1.5CH_2_Cl_2_
*M*_r_	916.05
Crystal system, space group	Triclinic, *P* 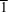
Temperature (K)	100
*a*, *b*, *c* (Å)	10.8268 (2), 12.5379 (2), 14.0896 (2)
α, β, γ (°)	87.421 (1), 86.988 (1), 77.089 (2)
*V* (Å^3^)	1860.58 (5)
*Z*	2
Radiation type	Mo *K*α
μ (mm^−1^)	3.90
Crystal size (mm)	0.23 × 0.15 × 0.05

Data collection	
Diffractometer	Rigaku XtaLAB Synergy-S
Absorption correction	Multi-scan (*CrysAlis PRO*; Rigaku OD, 2022 ▸)
*T*_min_, *T*_max_	0.743, 1.000
No. of measured, independent and observed [*I* > 2σ(*I*)] reflections	56836, 9245, 8573
*R*_int_	0.047
(sin θ/λ)_max_ (Å^−1^)	0.667

Refinement	
*R*[*F* ^2^ > 2σ(*F* ^2^)], *wR*(*F* ^2^), *S*	0.024, 0.059, 1.05
No. of reflections	9245
No. of parameters	535
No. of restraints	258
H-atom treatment	H-atom parameters constrained
Δρ_max_, Δρ_min_ (e Å^−3^)	1.04, −1.33

Computer programs: *CrysAlis PRO* (Rigaku OD, 2022 ▸), *OLEX2.solve* (Bourhis *et al.*, 2015 ▸), *SHELXL2018/3* (Sheldrick, 2015 ▸), *OLEX2* (Dolomanov *et al.*, 2009 ▸), and *publCIF* (Westrip, 2010 ▸).

## full crystallographic data

### Crystal data

**Table:**

[Ir(C_8_H_12_)(C_7_H_13_N_3_)(C_18_H_15_P)]BF_4_·1.5CH_2_Cl_2_	*Z* = 2
*M**_r_* = 916.05	*F*(000) = 910
Triclinic, *P*1	*D*_x_ = 1.635 Mg m^−^^3^
*a* = 10.8268 (2) Å	Mo *K*α radiation, λ = 0.71073 Å
*b* = 12.5379 (2) Å	Cell parameters from 39800 reflections
*c* = 14.0896 (2) Å	θ = 2.1–28.3°
α = 87.421 (1)°	µ = 3.90 mm^−^^1^
β = 86.988 (1)°	*T* = 100 K
γ = 77.089 (2)°	Plate, red
*V* = 1860.58 (5) Å^3^	0.23 × 0.15 × 0.05 mm

### Data collection

**Table:**

Rigaku XtaLAB Synergy-S diffractometer	8573 reflections with *I* > 2σ(*I*)
Detector resolution: 10.0 pixels mm^-1^	*R*_int_ = 0.047
ω scans	θ_max_ = 28.3°, θ_min_ = 1.9°
Absorption correction: multi-scan (CrysAlisPro; Rigaku OD, 2022)	*h* = −14→14
*T*_min_ = 0.743, *T*_max_ = 1.000	*k* = −16→16
56836 measured reflections	*l* = −18→18
9245 independent reflections	

### Refinement

**Table:**

Refinement on *F*^2^	Primary atom site location: iterative
Least-squares matrix: full	Hydrogen site location: inferred from neighbouring sites
*R*[*F*^2^ > 2σ(*F*^2^)] = 0.024	H-atom parameters constrained
*wR*(*F*^2^) = 0.059	*w* = 1/[σ^2^(*F*_o_^2^) + (0.0306*P*)^2^ + 1.9588*P*] where *P* = (*F*_o_^2^ + 2*F*_c_^2^)/3
*S* = 1.05	(Δ/σ)_max_ = 0.005
9245 reflections	Δρ_max_ = 1.04 e Å^−^^3^
535 parameters	Δρ_min_ = −1.32 e Å^−^^3^
258 restraints	

### Special details

**Table:**

Geometry. All esds (except the esd in the dihedral angle between two l.s. planes) are estimated using the full covariance matrix. The cell esds are taken into account individually in the estimation of esds in distances, angles and torsion angles; correlations between esds in cell parameters are only used when they are defined by crystal symmetry. An approximate (isotropic) treatment of cell esds is used for estimating esds involving l.s. planes.
Refinement. The H atoms were placed in claculated locations (C—H = 0.95–0.99 Å) and refined as riding atoms with *U*_iso_(H) = 1.2*U*_eq_(C) or 1.5*U*_eq_(methyl C).

### Fractional atomic coordinates and isotropic or equivalent isotropic displacement parameters (Å^2^)

**Table:**

	*x*	*y*	*z*	*U*_iso_*/*U*_eq_	Occ. (<1)
Ir1	0.42879 (2)	0.31295 (2)	0.83228 (2)	0.01084 (4)	
P1	0.46138 (6)	0.24673 (5)	0.67973 (5)	0.01209 (13)	
N1	0.4873 (2)	0.53500 (18)	0.77520 (16)	0.0137 (4)	
N2	0.5831 (2)	0.59119 (18)	0.75973 (17)	0.0172 (5)	
N3	0.6578 (2)	0.41849 (18)	0.80404 (15)	0.0135 (4)	
C1	0.5294 (2)	0.4294 (2)	0.80093 (17)	0.0122 (5)	
C2	0.6853 (3)	0.5177 (2)	0.7791 (2)	0.0170 (5)	
H2	0.768330	0.531266	0.776228	0.020*	
C3	0.3587 (3)	0.5900 (2)	0.7484 (2)	0.0174 (5)	
H3A	0.300273	0.540968	0.765245	0.021*	
H3B	0.358718	0.603485	0.678623	0.021*	
C4	0.3101 (3)	0.6974 (2)	0.7964 (2)	0.0214 (6)	
H4A	0.227615	0.733449	0.771804	0.032*	
H4B	0.370357	0.744625	0.783539	0.032*	
H4C	0.300708	0.683791	0.865200	0.032*	
C5	0.7507 (2)	0.3164 (2)	0.8281 (2)	0.0160 (5)	
H5	0.706314	0.254383	0.829472	0.019*	
C6	0.8591 (3)	0.2942 (3)	0.7526 (2)	0.0233 (6)	
H6A	0.824114	0.296898	0.689585	0.035*	
H6B	0.913142	0.221557	0.765008	0.035*	
H6C	0.909510	0.349854	0.754893	0.035*	
C7	0.8005 (3)	0.3224 (3)	0.9268 (2)	0.0300 (7)	
H7A	0.845658	0.382012	0.926709	0.045*	
H7B	0.858543	0.252941	0.943229	0.045*	
H7C	0.729154	0.336229	0.973736	0.045*	
C8	0.5887 (3)	0.2803 (2)	0.60086 (18)	0.0151 (5)	
C9	0.5887 (3)	0.3908 (2)	0.58311 (19)	0.0170 (5)	
H9	0.524697	0.445208	0.612714	0.020*	
C10	0.6805 (3)	0.4223 (3)	0.5230 (2)	0.0213 (6)	
H10	0.680084	0.497754	0.512465	0.026*	
C11	0.7729 (3)	0.3438 (3)	0.4781 (2)	0.0260 (7)	
H11	0.836610	0.365090	0.437482	0.031*	
C12	0.7720 (3)	0.2347 (3)	0.4928 (2)	0.0277 (7)	
H12	0.833794	0.180955	0.460532	0.033*	
C13	0.6813 (3)	0.2023 (3)	0.5544 (2)	0.0223 (6)	
H13	0.682573	0.126664	0.564848	0.027*	
C14	0.4928 (3)	0.0979 (2)	0.68138 (18)	0.0151 (5)	
C15	0.6038 (3)	0.0395 (2)	0.7234 (2)	0.0200 (6)	
H15	0.662608	0.078059	0.744960	0.024*	
C16	0.6284 (3)	−0.0734 (2)	0.7338 (2)	0.0234 (6)	
H16	0.703916	−0.112412	0.761865	0.028*	
C17	0.5414 (3)	−0.1292 (2)	0.7027 (2)	0.0238 (6)	
H17	0.558009	−0.206801	0.708941	0.029*	
C18	0.4307 (3)	−0.0723 (2)	0.6627 (2)	0.0241 (6)	
H18	0.371063	−0.110976	0.642770	0.029*	
C19	0.4061 (3)	0.0411 (2)	0.6514 (2)	0.0198 (6)	
H19	0.330383	0.079633	0.623336	0.024*	
C20	0.3257 (2)	0.2926 (2)	0.60536 (18)	0.0133 (5)	
C21	0.3355 (3)	0.2633 (2)	0.5099 (2)	0.0193 (6)	
H21	0.412847	0.220680	0.484384	0.023*	
C22	0.2324 (3)	0.2965 (3)	0.4526 (2)	0.0241 (6)	
H22	0.239487	0.276844	0.387901	0.029*	
C23	0.1186 (3)	0.3586 (3)	0.4898 (2)	0.0240 (6)	
H23	0.047804	0.380270	0.450726	0.029*	
C24	0.1087 (3)	0.3888 (2)	0.5836 (2)	0.0234 (6)	
H24	0.031145	0.431820	0.608514	0.028*	
C25	0.2116 (3)	0.3567 (2)	0.6418 (2)	0.0177 (5)	
H25	0.204365	0.378170	0.706068	0.021*	
C26	0.3710 (5)	0.1670 (7)	0.8993 (6)	0.0169 (11)	0.65
H26	0.408861	0.096875	0.867303	0.020*	0.65
C26*	0.4032 (12)	0.1559 (14)	0.8990 (13)	0.0170 (13)	0.35
H26*	0.440662	0.090734	0.860185	0.020*	0.35
C27	0.2664 (6)	0.2317 (5)	0.8550 (4)	0.0151 (10)	0.65
H27	0.242293	0.199930	0.796722	0.018*	0.65
C27*	0.2892 (14)	0.2125 (12)	0.8706 (10)	0.0182 (14)	0.35
H27*	0.260222	0.180920	0.814109	0.022*	0.35
C28	0.1581 (5)	0.3082 (5)	0.9061 (4)	0.0213 (9)	0.65
H28A	0.103175	0.264036	0.939870	0.026*	0.65
H28B	0.106647	0.356049	0.858195	0.026*	0.65
C28*	0.1784 (9)	0.2735 (8)	0.9343 (7)	0.0209 (12)	0.35
H28C	0.097464	0.269089	0.906701	0.025*	0.35
H28D	0.181611	0.236352	0.997880	0.025*	0.35
C29	0.1991 (5)	0.3808 (5)	0.9783 (4)	0.0193 (9)	0.65
H29A	0.134718	0.450504	0.982135	0.023*	0.65
H29B	0.201350	0.343581	1.041854	0.023*	0.65
C29*	0.1799 (10)	0.3920 (9)	0.9454 (7)	0.0207 (13)	0.35
H29C	0.129596	0.436617	0.895140	0.025*	0.35
H29D	0.138651	0.415765	1.007631	0.025*	0.35
C30	0.3295 (8)	0.4067 (9)	0.9534 (5)	0.0151 (11)	0.65
H30	0.327373	0.486865	0.947260	0.018*	0.65
C30*	0.3094 (17)	0.4132 (19)	0.9397 (12)	0.0175 (14)	0.35
H30*	0.309374	0.492744	0.930832	0.021*	0.35
C31	0.4450 (6)	0.3434 (8)	0.9842 (7)	0.0163 (11)	0.65
H31	0.508946	0.387536	0.995072	0.020*	0.65
C31*	0.4167 (14)	0.3534 (16)	0.9850 (14)	0.0171 (14)	0.35
H31*	0.477292	0.398121	1.002506	0.021*	0.35
C32	0.4657 (5)	0.2386 (4)	1.0399 (3)	0.0197 (9)	0.65
H32A	0.445303	0.253878	1.107962	0.024*	0.65
H32B	0.556294	0.201240	1.033323	0.024*	0.65
C32*	0.4137 (10)	0.2520 (9)	1.0527 (7)	0.0194 (12)	0.35
H32C	0.475830	0.248433	1.102566	0.023*	0.35
H32D	0.328457	0.261141	1.084544	0.023*	0.35
C33	0.3834 (5)	0.1614 (4)	1.0075 (3)	0.0194 (8)	0.65
H33A	0.421986	0.085219	1.028067	0.023*	0.65
H33B	0.297834	0.181703	1.038865	0.023*	0.65
C33*	0.4445 (10)	0.1451 (8)	1.0004 (6)	0.0191 (12)	0.35
H33C	0.537188	0.115216	1.000228	0.023*	0.35
H33D	0.402844	0.091744	1.035702	0.023*	0.35
Cl1	0.86248 (11)	0.00478 (10)	0.89852 (9)	0.0580 (3)	
Cl2	1.12218 (14)	−0.00307 (12)	0.82944 (14)	0.0864 (5)	
C34	1.0052 (5)	−0.0780 (4)	0.8554 (5)	0.0756 (17)	
H34A	1.036204	−0.136525	0.903530	0.091*	
H34B	0.989458	−0.113255	0.797146	0.091*	
Cl3	0.9176 (2)	−0.0652 (2)	0.43518 (18)	0.0604 (6)	0.5
Cl4	0.9699 (3)	0.0021 (2)	0.6212 (2)	0.0676 (7)	0.5
C35	0.9003 (9)	−0.0864 (8)	0.5585 (7)	0.059 (2)	0.5
H35A	0.940425	−0.163232	0.575832	0.071*	0.5
H35B	0.808940	−0.074099	0.577268	0.071*	0.5
F1	−0.0293 (15)	0.5305 (13)	0.7887 (8)	0.037 (2)	0.4
F1*	−0.0197 (11)	0.5456 (10)	0.7573 (6)	0.0390 (17)	0.6
F2	0.0497 (11)	0.6626 (8)	0.7107 (9)	0.043 (2)	0.4
F2*	0.0260 (7)	0.7081 (5)	0.7056 (5)	0.0344 (13)	0.6
F3	−0.15067 (19)	0.70529 (16)	0.79144 (15)	0.0359 (5)	
F4	0.0361 (2)	0.65703 (19)	0.86652 (14)	0.0397 (5)	
B1	−0.0248 (3)	0.6476 (3)	0.7842 (3)	0.0265 (8)	

### Atomic displacement parameters (Å^2^)

**Table:**

	*U*^11^	*U*^22^	*U*^33^	*U*^12^	*U*^13^	*U*^23^
Ir1	0.01229 (5)	0.01067 (5)	0.01045 (5)	−0.00460 (4)	0.00090 (3)	−0.00118 (3)
P1	0.0135 (3)	0.0110 (3)	0.0118 (3)	−0.0027 (2)	0.0000 (2)	−0.0013 (2)
N1	0.0145 (11)	0.0123 (10)	0.0155 (11)	−0.0051 (8)	−0.0009 (8)	−0.0005 (8)
N2	0.0169 (11)	0.0133 (11)	0.0232 (12)	−0.0076 (9)	0.0003 (9)	−0.0002 (9)
N3	0.0131 (10)	0.0129 (11)	0.0153 (11)	−0.0046 (8)	0.0007 (8)	−0.0005 (8)
C1	0.0134 (12)	0.0136 (12)	0.0095 (11)	−0.0022 (10)	0.0013 (9)	−0.0034 (9)
C2	0.0154 (13)	0.0143 (13)	0.0228 (14)	−0.0067 (10)	0.0012 (10)	−0.0015 (10)
C3	0.0171 (13)	0.0155 (13)	0.0194 (13)	−0.0025 (10)	−0.0051 (10)	0.0007 (10)
C4	0.0170 (14)	0.0189 (14)	0.0279 (15)	−0.0025 (11)	−0.0019 (11)	−0.0008 (11)
C5	0.0126 (12)	0.0122 (12)	0.0224 (14)	−0.0010 (10)	−0.0021 (10)	0.0018 (10)
C6	0.0181 (14)	0.0254 (15)	0.0229 (15)	0.0025 (12)	0.0003 (11)	−0.0032 (12)
C7	0.0268 (16)	0.0378 (19)	0.0199 (15)	0.0053 (14)	−0.0036 (12)	−0.0022 (13)
C8	0.0156 (13)	0.0199 (13)	0.0105 (12)	−0.0052 (10)	−0.0011 (9)	−0.0017 (10)
C9	0.0179 (13)	0.0200 (14)	0.0128 (12)	−0.0037 (11)	−0.0012 (10)	0.0002 (10)
C10	0.0226 (14)	0.0284 (16)	0.0153 (13)	−0.0116 (12)	−0.0038 (11)	0.0063 (11)
C11	0.0175 (14)	0.0454 (19)	0.0166 (14)	−0.0110 (13)	−0.0009 (11)	0.0040 (13)
C12	0.0186 (15)	0.0421 (19)	0.0193 (15)	−0.0006 (13)	0.0043 (11)	−0.0056 (13)
C13	0.0216 (14)	0.0238 (15)	0.0195 (14)	−0.0001 (12)	0.0012 (11)	−0.0058 (11)
C14	0.0199 (13)	0.0121 (12)	0.0131 (12)	−0.0027 (10)	−0.0005 (10)	−0.0019 (9)
C15	0.0229 (14)	0.0160 (14)	0.0207 (14)	−0.0032 (11)	−0.0037 (11)	−0.0002 (11)
C16	0.0272 (16)	0.0197 (14)	0.0197 (14)	0.0020 (12)	−0.0004 (12)	0.0019 (11)
C17	0.0371 (17)	0.0118 (13)	0.0210 (14)	−0.0037 (12)	0.0064 (12)	−0.0016 (11)
C18	0.0362 (17)	0.0168 (14)	0.0221 (15)	−0.0119 (13)	−0.0015 (12)	−0.0024 (11)
C19	0.0256 (15)	0.0163 (13)	0.0173 (13)	−0.0038 (11)	−0.0027 (11)	−0.0004 (10)
C20	0.0149 (12)	0.0110 (12)	0.0145 (12)	−0.0041 (10)	−0.0017 (9)	0.0016 (9)
C21	0.0167 (13)	0.0224 (14)	0.0170 (13)	−0.0002 (11)	−0.0013 (10)	−0.0018 (11)
C22	0.0240 (15)	0.0318 (17)	0.0158 (14)	−0.0036 (13)	−0.0043 (11)	−0.0020 (12)
C23	0.0170 (14)	0.0296 (16)	0.0256 (15)	−0.0053 (12)	−0.0076 (11)	0.0064 (12)
C24	0.0151 (13)	0.0236 (15)	0.0299 (16)	−0.0020 (11)	0.0020 (11)	0.0025 (12)
C25	0.0172 (13)	0.0156 (13)	0.0204 (13)	−0.0050 (10)	0.0021 (10)	0.0010 (10)
C26	0.020 (3)	0.019 (2)	0.0149 (16)	−0.011 (2)	−0.001 (2)	−0.0004 (15)
C26*	0.019 (3)	0.020 (2)	0.015 (2)	−0.010 (3)	−0.003 (3)	0.003 (2)
C27	0.015 (2)	0.021 (2)	0.013 (2)	−0.0122 (18)	−0.0021 (16)	0.0018 (16)
C27*	0.019 (3)	0.022 (3)	0.015 (2)	−0.008 (2)	−0.001 (2)	−0.001 (2)
C28	0.0175 (18)	0.030 (2)	0.0171 (19)	−0.0084 (17)	−0.0009 (16)	0.0064 (16)
C28*	0.018 (2)	0.027 (3)	0.018 (3)	−0.006 (2)	0.000 (2)	0.003 (2)
C29	0.0170 (19)	0.0257 (19)	0.014 (2)	−0.0031 (16)	0.0018 (17)	−0.0017 (18)
C29*	0.018 (2)	0.027 (2)	0.015 (3)	−0.002 (2)	0.000 (2)	0.000 (2)
C30	0.015 (3)	0.0189 (19)	0.012 (2)	−0.0059 (19)	0.0013 (17)	−0.0042 (18)
C30*	0.018 (3)	0.022 (2)	0.012 (3)	−0.003 (2)	−0.001 (2)	−0.005 (2)
C31	0.017 (3)	0.022 (2)	0.0106 (15)	−0.005 (2)	−0.002 (2)	−0.0024 (15)
C31*	0.019 (3)	0.021 (2)	0.012 (2)	−0.006 (3)	−0.001 (3)	−0.003 (2)
C32	0.022 (2)	0.0242 (18)	0.0134 (16)	−0.0053 (19)	−0.0022 (17)	0.0012 (14)
C32*	0.020 (3)	0.023 (2)	0.014 (2)	−0.004 (3)	−0.002 (2)	0.001 (2)
C33	0.022 (2)	0.0211 (18)	0.0158 (16)	−0.0069 (18)	−0.0009 (17)	0.0033 (14)
C33*	0.021 (3)	0.022 (2)	0.014 (2)	−0.006 (2)	−0.003 (2)	0.0038 (19)
Cl1	0.0514 (6)	0.0527 (6)	0.0722 (8)	−0.0151 (5)	−0.0070 (5)	−0.0036 (6)
Cl2	0.0627 (8)	0.0624 (8)	0.1313 (14)	−0.0159 (7)	0.0148 (9)	0.0144 (8)
C34	0.064 (3)	0.031 (2)	0.130 (5)	−0.011 (2)	0.010 (3)	0.007 (3)
Cl3	0.0549 (13)	0.0673 (15)	0.0593 (14)	−0.0123 (11)	−0.0103 (11)	−0.0030 (11)
Cl4	0.0732 (17)	0.0561 (14)	0.0708 (16)	−0.0035 (12)	−0.0137 (13)	−0.0154 (12)
C35	0.047 (5)	0.060 (6)	0.072 (7)	−0.013 (4)	−0.001 (5)	−0.015 (5)
F1	0.023 (4)	0.027 (4)	0.062 (7)	−0.005 (3)	−0.007 (5)	−0.017 (5)
F1*	0.035 (3)	0.033 (3)	0.047 (4)	−0.002 (2)	0.003 (3)	−0.013 (3)
F2	0.032 (4)	0.076 (6)	0.027 (3)	−0.023 (5)	0.003 (3)	−0.005 (5)
F2*	0.032 (3)	0.058 (4)	0.0184 (18)	−0.023 (3)	0.0040 (19)	0.003 (3)
F3	0.0325 (11)	0.0314 (11)	0.0401 (11)	−0.0016 (8)	0.0078 (9)	−0.0008 (9)
F4	0.0430 (12)	0.0611 (14)	0.0251 (10)	−0.0329 (11)	−0.0002 (9)	−0.0033 (9)
B1	0.0208 (17)	0.039 (2)	0.0243 (17)	−0.0156 (15)	0.0060 (13)	−0.0119 (15)

### Geometric parameters (Å, º)

**Table:**

Ir1—P1	2.3203 (6)	C22—H22	0.9500
Ir1—C1	2.029 (3)	C22—C23	1.392 (4)
Ir1—C26	2.219 (9)	C23—H23	0.9500
Ir1—C26*	2.210 (18)	C23—C24	1.384 (4)
Ir1—C27	2.223 (7)	C24—H24	0.9500
Ir1—C27*	2.206 (16)	C24—C25	1.394 (4)
Ir1—C30	2.208 (9)	C25—H25	0.9500
Ir1—C30*	2.186 (19)	C26—H26	1.0000
Ir1—C31	2.213 (10)	C26—C27	1.396 (8)
Ir1—C31*	2.22 (2)	C26—C33	1.534 (9)
P1—C8	1.836 (3)	C26*—H26*	1.0000
P1—C14	1.820 (3)	C26*—C27*	1.350 (15)
P1—C20	1.821 (3)	C26*—C33*	1.511 (18)
N1—N2	1.382 (3)	C27—H27	1.0000
N1—C1	1.340 (3)	C27—C28	1.511 (8)
N1—C3	1.470 (3)	C27*—H27*	1.0000
N2—C2	1.304 (4)	C27*—C28*	1.538 (16)
N3—C1	1.368 (3)	C28—H28A	0.9900
N3—C2	1.369 (3)	C28—H28B	0.9900
N3—C5	1.479 (3)	C28—C29	1.541 (8)
C2—H2	0.9500	C28*—H28C	0.9900
C3—H3A	0.9900	C28*—H28D	0.9900
C3—H3B	0.9900	C28*—C29*	1.505 (16)
C3—C4	1.508 (4)	C29—H29A	0.9900
C4—H4A	0.9800	C29—H29B	0.9900
C4—H4B	0.9800	C29—C30	1.537 (10)
C4—H4C	0.9800	C29*—H29C	0.9900
C5—H5	1.0000	C29*—H29D	0.9900
C5—C6	1.528 (4)	C29*—C30*	1.48 (2)
C5—C7	1.528 (4)	C30—H30	1.0000
C6—H6A	0.9800	C30—C31	1.402 (8)
C6—H6B	0.9800	C30*—H30*	1.0000
C6—H6C	0.9800	C30*—C31*	1.402 (18)
C7—H7A	0.9800	C31—H31	1.0000
C7—H7B	0.9800	C31—C32	1.479 (10)
C7—H7C	0.9800	C31*—H31*	1.0000
C8—C9	1.397 (4)	C31*—C32*	1.56 (2)
C8—C13	1.393 (4)	C32—H32A	0.9900
C9—H9	0.9500	C32—H32B	0.9900
C9—C10	1.385 (4)	C32—C33	1.551 (7)
C10—H10	0.9500	C32*—H32C	0.9900
C10—C11	1.385 (4)	C32*—H32D	0.9900
C11—H11	0.9500	C32*—C33*	1.522 (14)
C11—C12	1.376 (5)	C33—H33A	0.9900
C12—H12	0.9500	C33—H33B	0.9900
C12—C13	1.393 (4)	C33*—H33C	0.9900
C13—H13	0.9500	C33*—H33D	0.9900
C14—C15	1.404 (4)	Cl1—C34	1.753 (5)
C14—C19	1.390 (4)	Cl2—C34	1.753 (5)
C15—H15	0.9500	C34—H34A	0.9900
C15—C16	1.383 (4)	C34—H34B	0.9900
C16—H16	0.9500	Cl3—C35	1.752 (11)
C16—C17	1.391 (4)	Cl4—C35	1.764 (9)
C17—H17	0.9500	C35—H35A	0.9900
C17—C18	1.384 (5)	C35—H35B	0.9900
C18—H18	0.9500	F1—B1	1.478 (16)
C18—C19	1.390 (4)	F1*—B1	1.339 (13)
C19—H19	0.9500	F2—B1	1.311 (13)
C20—C21	1.402 (4)	F2*—B1	1.466 (8)
C20—C25	1.401 (4)	F3—B1	1.394 (4)
C21—H21	0.9500	F4—B1	1.387 (4)
C21—C22	1.388 (4)		
			
C1—Ir1—P1	92.69 (7)	C23—C24—H24	119.7
C1—Ir1—C26	160.8 (2)	C23—C24—C25	120.5 (3)
C1—Ir1—C26*	153.1 (4)	C25—C24—H24	119.7
C1—Ir1—C27	161.03 (17)	C20—C25—H25	120.1
C1—Ir1—C27*	169.3 (4)	C24—C25—C20	119.8 (3)
C1—Ir1—C30	90.8 (3)	C24—C25—H25	120.1
C1—Ir1—C30*	92.2 (6)	Ir1—C26—H26	114.4
C1—Ir1—C31	87.4 (3)	C27—C26—Ir1	71.8 (4)
C1—Ir1—C31*	90.2 (5)	C27—C26—H26	114.4
C26—Ir1—P1	95.9 (2)	C27—C26—C33	122.7 (6)
C26—Ir1—C27	36.63 (19)	C33—C26—Ir1	111.9 (5)
C26*—Ir1—P1	94.3 (5)	C33—C26—H26	114.4
C26*—Ir1—C31*	79.7 (7)	Ir1—C26*—H26*	114.0
C27—Ir1—P1	89.18 (18)	C27*—C26*—Ir1	72.0 (9)
C27*—Ir1—P1	92.1 (4)	C27*—C26*—H26*	114.0
C27*—Ir1—C26*	35.6 (4)	C27*—C26*—C33*	124.8 (12)
C27*—Ir1—C31*	86.4 (6)	C33*—C26*—Ir1	110.5 (10)
C30—Ir1—P1	158.19 (17)	C33*—C26*—H26*	114.0
C30—Ir1—C26	87.6 (3)	Ir1—C27—H27	114.5
C30—Ir1—C27	80.8 (3)	C26—C27—Ir1	71.5 (4)
C30—Ir1—C31	37.0 (2)	C26—C27—H27	114.5
C30*—Ir1—P1	150.5 (4)	C26—C27—C28	124.5 (6)
C30*—Ir1—C26*	94.4 (7)	C28—C27—Ir1	109.1 (4)
C30*—Ir1—C27*	79.0 (7)	C28—C27—H27	114.5
C30*—Ir1—C31*	37.1 (4)	Ir1—C27*—H27*	112.8
C31—Ir1—P1	164.72 (18)	C26*—C27*—Ir1	72.4 (10)
C31—Ir1—C26	79.8 (4)	C26*—C27*—H27*	112.8
C31—Ir1—C27	95.7 (3)	C26*—C27*—C28*	126.9 (12)
C31*—Ir1—P1	171.5 (5)	C28*—C27*—Ir1	112.1 (8)
C8—P1—Ir1	119.96 (9)	C28*—C27*—H27*	112.8
C14—P1—Ir1	111.55 (9)	C27—C28—H28A	108.6
C14—P1—C8	104.04 (12)	C27—C28—H28B	108.6
C14—P1—C20	105.26 (12)	C27—C28—C29	114.6 (4)
C20—P1—Ir1	113.78 (9)	H28A—C28—H28B	107.6
C20—P1—C8	100.68 (12)	C29—C28—H28A	108.6
N2—N1—C3	118.2 (2)	C29—C28—H28B	108.6
C1—N1—N2	113.4 (2)	C27*—C28*—H28C	108.9
C1—N1—C3	127.7 (2)	C27*—C28*—H28D	108.9
C2—N2—N1	103.5 (2)	H28C—C28*—H28D	107.7
C1—N3—C2	108.2 (2)	C29*—C28*—C27*	113.3 (9)
C1—N3—C5	125.7 (2)	C29*—C28*—H28C	108.9
C2—N3—C5	126.0 (2)	C29*—C28*—H28D	108.9
N1—C1—Ir1	128.93 (19)	C28—C29—H29A	108.8
N1—C1—N3	103.5 (2)	C28—C29—H29B	108.8
N3—C1—Ir1	127.53 (19)	H29A—C29—H29B	107.7
N2—C2—N3	111.4 (2)	C30—C29—C28	113.7 (5)
N2—C2—H2	124.3	C30—C29—H29A	108.8
N3—C2—H2	124.3	C30—C29—H29B	108.8
N1—C3—H3A	109.0	C28*—C29*—H29C	108.9
N1—C3—H3B	109.0	C28*—C29*—H29D	108.9
N1—C3—C4	113.0 (2)	H29C—C29*—H29D	107.7
H3A—C3—H3B	107.8	C30*—C29*—C28*	113.2 (11)
C4—C3—H3A	109.0	C30*—C29*—H29C	108.9
C4—C3—H3B	109.0	C30*—C29*—H29D	108.9
C3—C4—H4A	109.5	Ir1—C30—H30	113.7
C3—C4—H4B	109.5	C29—C30—Ir1	112.3 (5)
C3—C4—H4C	109.5	C29—C30—H30	113.7
H4A—C4—H4B	109.5	C31—C30—Ir1	71.7 (6)
H4A—C4—H4C	109.5	C31—C30—C29	124.6 (7)
H4B—C4—H4C	109.5	C31—C30—H30	113.7
N3—C5—H5	108.2	Ir1—C30*—H30*	112.8
N3—C5—C6	110.7 (2)	C29*—C30*—Ir1	110.9 (11)
N3—C5—C7	110.3 (2)	C29*—C30*—H30*	112.8
C6—C5—H5	108.2	C31*—C30*—Ir1	72.9 (11)
C6—C5—C7	111.2 (2)	C31*—C30*—C29*	127.5 (15)
C7—C5—H5	108.2	C31*—C30*—H30*	112.8
C5—C6—H6A	109.5	Ir1—C31—H31	113.4
C5—C6—H6B	109.5	C30—C31—Ir1	71.3 (5)
C5—C6—H6C	109.5	C30—C31—H31	113.4
H6A—C6—H6B	109.5	C30—C31—C32	127.4 (7)
H6A—C6—H6C	109.5	C32—C31—Ir1	109.8 (5)
H6B—C6—H6C	109.5	C32—C31—H31	113.4
C5—C7—H7A	109.5	Ir1—C31*—H31*	114.5
C5—C7—H7B	109.5	C30*—C31*—Ir1	70.0 (11)
C5—C7—H7C	109.5	C30*—C31*—H31*	114.5
H7A—C7—H7B	109.5	C30*—C31*—C32*	122.9 (13)
H7A—C7—H7C	109.5	C32*—C31*—Ir1	112.7 (10)
H7B—C7—H7C	109.5	C32*—C31*—H31*	114.5
C9—C8—P1	117.8 (2)	C31—C32—H32A	109.1
C13—C8—P1	123.7 (2)	C31—C32—H32B	109.1
C13—C8—C9	118.4 (3)	C31—C32—C33	112.5 (5)
C8—C9—H9	119.5	H32A—C32—H32B	107.8
C10—C9—C8	121.0 (3)	C33—C32—H32A	109.1
C10—C9—H9	119.5	C33—C32—H32B	109.1
C9—C10—H10	120.0	C31*—C32*—H32C	109.1
C9—C10—C11	120.0 (3)	C31*—C32*—H32D	109.1
C11—C10—H10	120.0	H32C—C32*—H32D	107.9
C10—C11—H11	120.2	C33*—C32*—C31*	112.4 (9)
C12—C11—C10	119.7 (3)	C33*—C32*—H32C	109.1
C12—C11—H11	120.2	C33*—C32*—H32D	109.1
C11—C12—H12	119.7	C26—C33—C32	112.3 (4)
C11—C12—C13	120.7 (3)	C26—C33—H33A	109.2
C13—C12—H12	119.7	C26—C33—H33B	109.2
C8—C13—C12	120.3 (3)	C32—C33—H33A	109.2
C8—C13—H13	119.9	C32—C33—H33B	109.2
C12—C13—H13	119.9	H33A—C33—H33B	107.9
C15—C14—P1	117.8 (2)	C26*—C33*—C32*	114.2 (9)
C19—C14—P1	122.6 (2)	C26*—C33*—H33C	108.7
C19—C14—C15	119.3 (3)	C26*—C33*—H33D	108.7
C14—C15—H15	119.6	C32*—C33*—H33C	108.7
C16—C15—C14	120.8 (3)	C32*—C33*—H33D	108.7
C16—C15—H15	119.6	H33C—C33*—H33D	107.6
C15—C16—H16	120.3	Cl1—C34—H34A	109.2
C15—C16—C17	119.3 (3)	Cl1—C34—H34B	109.2
C17—C16—H16	120.3	Cl2—C34—Cl1	112.1 (3)
C16—C17—H17	119.8	Cl2—C34—H34A	109.2
C18—C17—C16	120.3 (3)	Cl2—C34—H34B	109.2
C18—C17—H17	119.8	H34A—C34—H34B	107.9
C17—C18—H18	119.7	Cl3—C35—Cl4	111.7 (6)
C17—C18—C19	120.5 (3)	Cl3—C35—H35A	109.3
C19—C18—H18	119.7	Cl3—C35—H35B	109.3
C14—C19—H19	120.1	Cl4—C35—H35A	109.3
C18—C19—C14	119.8 (3)	Cl4—C35—H35B	109.3
C18—C19—H19	120.1	H35A—C35—H35B	107.9
C21—C20—P1	119.5 (2)	F1*—B1—F2*	108.8 (5)
C25—C20—P1	121.1 (2)	F1*—B1—F3	109.8 (6)
C25—C20—C21	119.4 (2)	F1*—B1—F4	116.2 (5)
C20—C21—H21	119.9	F2—B1—F1	107.2 (7)
C22—C21—C20	120.2 (3)	F2—B1—F3	121.4 (5)
C22—C21—H21	119.9	F2—B1—F4	108.6 (6)
C21—C22—H22	119.9	F3—B1—F1	105.8 (7)
C21—C22—C23	120.1 (3)	F3—B1—F2*	101.5 (4)
C23—C22—H22	119.9	F4—B1—F1	102.0 (6)
C22—C23—H23	120.0	F4—B1—F2*	109.4 (4)
C24—C23—C22	120.0 (3)	F4—B1—F3	110.1 (3)
C24—C23—H23	120.0		
			
Ir1—P1—C8—C9	−56.0 (2)	C9—C10—C11—C12	0.8 (4)
Ir1—P1—C8—C13	127.2 (2)	C10—C11—C12—C13	−1.9 (5)
Ir1—P1—C14—C15	−64.4 (2)	C11—C12—C13—C8	1.1 (5)
Ir1—P1—C14—C19	109.2 (2)	C13—C8—C9—C10	−1.9 (4)
Ir1—P1—C20—C21	176.48 (19)	C14—P1—C8—C9	178.4 (2)
Ir1—P1—C20—C25	−3.5 (2)	C14—P1—C8—C13	1.6 (3)
Ir1—C26—C27—C28	−100.9 (6)	C14—P1—C20—C21	−61.1 (2)
Ir1—C26—C33—C32	−15.5 (5)	C14—P1—C20—C25	119.0 (2)
Ir1—C26*—C27*—C28*	−104.8 (14)	C14—C15—C16—C17	−0.5 (4)
Ir1—C26*—C33*—C32*	36.6 (11)	C15—C14—C19—C18	−0.5 (4)
Ir1—C27—C28—C29	−34.4 (5)	C15—C16—C17—C18	−0.7 (4)
Ir1—C27*—C28*—C29*	7.8 (11)	C16—C17—C18—C19	1.2 (5)
Ir1—C30—C31—C32	−101.0 (9)	C17—C18—C19—C14	−0.6 (4)
Ir1—C30*—C31*—C32*	−104.7 (15)	C19—C14—C15—C16	1.0 (4)
Ir1—C31—C32—C33	−40.6 (5)	C20—P1—C8—C9	69.6 (2)
Ir1—C31*—C32*—C33*	10.7 (12)	C20—P1—C8—C13	−107.2 (2)
P1—C8—C9—C10	−178.8 (2)	C20—P1—C14—C15	171.7 (2)
P1—C8—C13—C12	177.5 (2)	C20—P1—C14—C19	−14.7 (3)
P1—C14—C15—C16	174.9 (2)	C20—C21—C22—C23	−0.3 (5)
P1—C14—C19—C18	−174.0 (2)	C21—C20—C25—C24	1.2 (4)
P1—C20—C21—C22	179.2 (2)	C21—C22—C23—C24	1.0 (5)
P1—C20—C25—C24	−178.8 (2)	C22—C23—C24—C25	−0.6 (5)
N1—N2—C2—N3	1.1 (3)	C23—C24—C25—C20	−0.5 (4)
N2—N1—C1—Ir1	179.62 (18)	C25—C20—C21—C22	−0.8 (4)
N2—N1—C1—N3	1.4 (3)	C26—C27—C28—C29	45.9 (8)
N2—N1—C3—C4	−57.1 (3)	C26*—C27*—C28*—C29*	91.9 (16)
C1—N1—N2—C2	−1.6 (3)	C27—C26—C33—C32	−97.4 (7)
C1—N1—C3—C4	133.5 (3)	C27—C28—C29—C30	28.6 (7)
C1—N3—C2—N2	−0.3 (3)	C27*—C26*—C33*—C32*	−45.2 (18)
C1—N3—C5—C6	129.0 (3)	C27*—C28*—C29*—C30*	−30.3 (13)
C1—N3—C5—C7	−107.5 (3)	C28—C29—C30—Ir1	−7.8 (6)
C2—N3—C1—Ir1	−178.91 (18)	C28—C29—C30—C31	−90.5 (9)
C2—N3—C1—N1	−0.6 (3)	C28*—C29*—C30*—Ir1	38.1 (13)
C2—N3—C5—C6	−49.5 (3)	C28*—C29*—C30*—C31*	−46 (2)
C2—N3—C5—C7	74.0 (3)	C29—C30—C31—Ir1	105.0 (8)
C3—N1—N2—C2	−172.5 (2)	C29—C30—C31—C32	4.0 (13)
C3—N1—C1—Ir1	−10.5 (4)	C29*—C30*—C31*—Ir1	103.5 (18)
C3—N1—C1—N3	171.2 (2)	C29*—C30*—C31*—C32*	−1 (3)
C5—N3—C1—Ir1	2.4 (4)	C30—C31—C32—C33	40.5 (10)
C5—N3—C1—N1	−179.4 (2)	C30*—C31*—C32*—C33*	90.9 (17)
C5—N3—C2—N2	178.4 (2)	C31—C32—C33—C26	37.6 (7)
C8—P1—C14—C15	66.3 (2)	C31*—C32*—C33*—C26*	−31.3 (13)
C8—P1—C14—C19	−120.1 (2)	C33—C26—C27—Ir1	104.8 (7)
C8—P1—C20—C21	46.8 (2)	C33—C26—C27—C28	3.9 (10)
C8—P1—C20—C25	−133.1 (2)	C33*—C26*—C27*—Ir1	102.8 (14)
C8—C9—C10—C11	1.1 (4)	C33*—C26*—C27*—C28*	−2 (2)
C9—C8—C13—C12	0.7 (4)		

### Hydrogen-bond geometry (Å, º)

**Table:**

*D*—H···*A*	*D*—H	H···*A*	*D*···*A*	*D*—H···*A*
C2—H2···F1^i^	0.95	2.21	3.140 (16)	168
C11—H11···F2^ii^	0.95	2.37	3.192 (13)	145

Symmetry codes: (i) *x*+1, *y*, *z*; (ii) −*x*+1, −*y*+1, −*z*+1.
